# Protocol for stable isotopic tracing to assess cellular lipogenic activity in induced neural stem cells

**DOI:** 10.1016/j.xpro.2025.104309

**Published:** 2026-01-03

**Authors:** Rosana-Bristena Ionescu, Julie A. Reisz, Monika Dzieciatkowska, Daniel Stephenson, Alexandra M. Nicaise, Pranathi Prasad, Cory M. Willis, Marta Suarez Cubero, Luca Peruzzotti-Jametti, Frank Edenhofer, Christian Frezza, Stefano Pluchino, Angelo D’Alessandro

**Affiliations:** 1 Department of Clinical Neurosciences and NIHR Biomedical Research Centre, University of Cambridge, Cambridge CB2 0AH, UK; 2 Department of Biochemistry and Molecular Genetics, University of Colorado Anschutz, Aurora, CO 80045, USA; 3 Genomics, Stem Cell Biology and Regenerative Medicine Group, Institute of Molecular Biology & CMBI, Leopold-Franzens-University Innsbruck, Innsbruck 6020, Austria; 4 Department of Metabolism, Digestion and Reproduction, Imperial College London, London W12 0NN, UK; 5 University of Cologne, Faculty of Medicine and University Hospital Cologne, Institute for Metabolomics in Ageing, Cluster of Excellence Cellular Stress Responses in Aging-associated Diseases (CECAD), 50931 Cologne, Germany; 6 Univrsity of Cologne, Faculty of Mathematics and Natural Sciences, Institute for Genetics, Cluster of Excellence Cellular Stress Responses in Aging-associated Diseases (CECAD), 50931 Cologne, Germany

**Keywords:** metabolism, neuroscience, proteomics, stem cells, protocols in metabolomics and lipidomics

## Abstract

Here, we present a protocol to assess the lipogenic phenotype of induced neural stem cells (iNSCs) using stable isotopic tracing. We describe steps for the culture and preparation of iNSCs, labeling with [^13^C_6_]-glucose and [^13^C_5_, ^15^N_2_]-glutamine, and the subsequent extraction of metabolites, lipids, and proteins from the same sample. This protocol supports single-specimen, mass spectrometry-based multi-omics workflows and is applicable to steady-state analyses, stable isotope tracing, and characterization of protein post-translational modifications.

For complete details on the use and execution of this protocol, please refer to Ionescu et al.^1^

## Highlights

• Instructions for culturing and preparing iNSCs for multi-omics profiling
• Steps for stable isotopic labeling with [^13^C_6_]-glucose and [^13^C_5_,^15^N_2_]-glutamine
• How to sequentially extract metabolites, lipids, and proteins from one sample
• Guidance on UHPLC-MS data acquisition and analysis

## Abstract (editor highlights)

Publisher’s note: Undertaking any experimental protocol requires adherence to local institutional guidelines for laboratory safety and ethics.

## Graphical abstract

**Figure:**
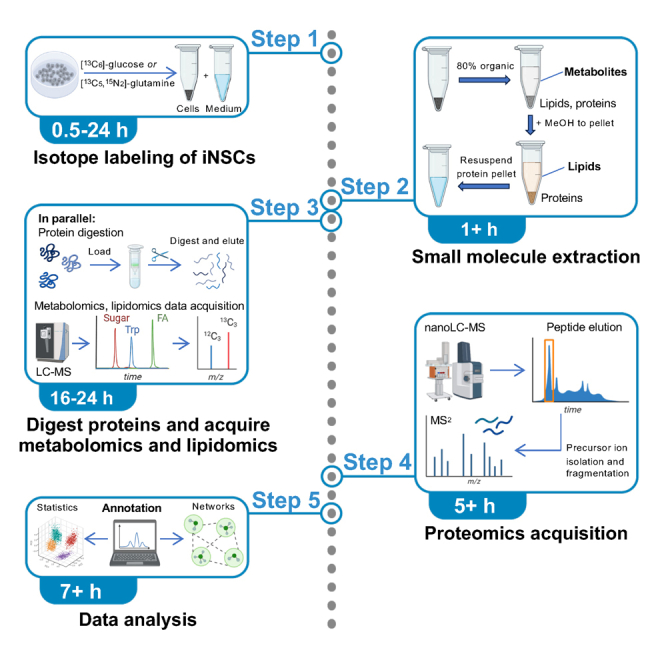

## Before you begin

Mass spectrometry (MS) has been established as a gold standard tool in omics analysis due to its high sensitivity, robustness, and adaptability for wide-ranging biomedical applications. These features are leveraged to rapidly collect hundreds to thousands of data points on unique molecular features in short, clinically relevant time frames. Similar to other analytical technologies, the evolution of MS omics is marked by improving sensitivity that allows low-input samples to yield broad and rich data sets. In addition to improved sensitivity offered by state-of-the-art instrumentation, we have developed tandem-in-time workflows for the generation of metabolomics, lipidomics, and proteomics (herein referred to as “multiomics”) data from single specimens at a one-stop academic facility. This approach is successful with low input samples and minimizes the time between omics analyses, maintaining sample integrity and maximizing congruity among the various acquired data sets. Of these 3 omics, sample preparation for proteomics is performed subsequent to metabolomics, lipidomics, or both. Herein we describe options for starting points of metabolomics, lipidomics, or both omics prior to proteomics so that the analyses can be tailored to specific research needs.

### Innovation

**Figure 1:**
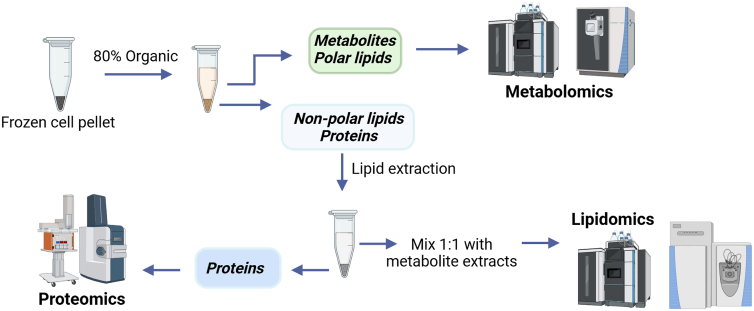
Overview of single specimen workflow for metabolomics followed by lipidomics and/or proteomics

This protocol integrates stable isotopic tracing with a streamlined, single-specimen multiomics workflow to characterize lipogenic activity in iNSCs (Figure 1). Unlike conventional approaches that require separate cultures for metabolomics, lipidomics, and proteomics, our method enables sequential extraction of metabolites, lipids, and proteins from the same biological specimen, preserving sample integrity and ensuring cross-omics comparability. The workflow is optimized for iNSC cultures yielding low amounts of extractable protein and metabolites, using stepwise extraction methods compatible with ultra-high-performance liquid chromatography-mass spectrometry (UHPLC-MS) platforms.

## Key resources table

**Table:** 

REAGENT or RESOURCE	SOURCE	IDENTIFIER
**Experimental models: Cell lines**		

iNSC	This study and Ionescu et al.^1^	N/A

**Chemicals, peptides, and recombinant proteins**		

Bovine Serum Albumin	Thermo Fisher Scientific	Cat#1002096887
DMEM/F12	Thermo Fisher Scientific	Cat#11320033
DMEM/F12 w/o glucose	Elabscience	Cat#EP-CM-L0127-ELA
DMEM/F12 w/o glutamine	Thermo Fisher Scientific	Cat#21331020
Neurobasal A Medium	Thermo Fisher Scientific	Cat#21103049
GlutaMAX	Thermo Fisher Scientific	Cat#35050038
B-27 Supplement	Thermo Fisher Scientific	Cat#17504044
*N*-2 Supplement	Thermo Fisher Scientific	Cat#17502001
CHIR99021	Cell Guidance Systems	Cat#SM13
hLIF	Cell Signaling Technology	Cat#62226S
SB-431542	Cayman Chemicals	Cat#13031
Y-27632	Miltenyi Biotec	Cat#130-103-933
Growth Factor Reduced Matrigel Matrix	Corning	Cat#354230
Accutase	Thermo Fisher Scientific	Cat#A1110501
XF 1.0 M glucose solution	Agilent	Cat#103577-100
XF 100 mM pyruvate solution	Agilent	Cat#103578-100
D-Glucose-^13^C_6_	Thermo Fisher Scientific	Cat#389374
L-Glutamine-^13^C_5_,^15^N_2_	Thermo Fisher Scientific	Cat#607983
2-Mercaptoethanol	Thermo Fisher Scientific	Cat#21985023
DMSO	Sigma-Aldrich	Cat#D2650
Acetic Acid	Carolina	Cat#841289
KnockOut Serum Replacement	Thermo Fisher Scientific	Cat#10828028
Acetonitrile	Fisher Scientific	Cat#A955
Methanol	Fisher Scientific	Cat# A456
Water	Fisher Scientific	Cat#W64
Water with 0.1% formic acid, Optima LC/MS	Fisher Scientific	Cat# LS118
Acetonitrile with 0.1% formic acid, Optima LC/MS	Fisher Scientific	Cat# LS120
80% Acetonitrile, 20% Water with 0.1% formic acid, Optima LC/MS	Fisher Scientific	Cat# LS122500
Sodium dodecyl sulfate	Sigma-Aldrich	Cat#L3771
Triethylammonium bicarbonate (1 M)	Sigma-Aldrich	Cat# 18597
Dithiothreitol	Sigma-Aldrich	Cat#D9779
Iodoacetamide	Sigma-Aldrich	Cat#I1149
Ammonium bicarbonate	Sigma-Aldrich	Cat#09830
Urea	Sigma-Aldrich	Cat# U5378
Phosphoric acid	Sigma-Aldrich	Cat#452289
Sequencing grade trypsin	Promega	Cat#V5113
Isopropanol	Fisher Scientific	Cat# A461
Calibration solutions	Fisher Scientific	Cat#PI88323 and PI88324

**Software and algorithms**		

Evosep One - timsTOF Pro	Bruker	N/A
Vanquish UHPLC - Q Exactive	Thermo Fisher Scientific	N/A
MS Convert	ProteoWizard	N/A
Scaffold	Proteome Software	N/A
FragPipe	Nesvizhskii lab	https://fragpipe.nesvilab.org/
MSFragger	Nesvizhskii lab	https://msfragger.nesvilab.org/
El-Maven	Elucidata	https://docs.polly.elucidata.io/Apps/Metabolomic%20Data/El-MAVEN.html
RawConverter	Yates lab	https://github.com/proteomicsyates/RawConverter
LipidSearch v.5.1	Thermo Fisher Scientific	Cat# OPTON-30879
MetaboAnalyst	–	https://www.metaboanalyst.ca/home.xhtml

**Other**		

Evosep One - timsTOF Pro	Bruker	N/A
Graduated cylinder, glass, 1L	Fisher Scientific	Cat#08-549-11F
Bottle - glass, with cap, 1 L	Fisher Scientific	Cat#06-414-1D
Metabolomics LC columns - ACQUITY BEH C18, 2.1 × 100 mm, 1.7 um	Waters	Cat#186002352
Conical centrifuge tubes, 50 mL	Fisher Scientific	Cat#14-432-22
Microcentrifuge tubes, 1.7 mL	Axygen	Cat#MCT175LC
Refrigerated centrifuge	Eppendorf	Cat#022620659
S-Trap micro spin columns	Protifi	Cat#C02-micro
Vivacon 500 spin filters, 10k MWCO	Fisher Scientific	Cat#14558344
Autosampler vials	Phenomenex	Cat#AR0-39P2-13
Caps for autosampler vials	Phenomenex	Cat#AR0-8972-13
SpeedVac	Labconco	Cat#7983013
Pierce C18 spin tips	Thermo Scientific	Cat#84850
Evotips Pure	EvoSep	EV2013

## Materials and equipment

### CHIR99021 stock (10 mM)

Dilute 10 mg CHIR99021 in 2,150 μL of DMSO.

***Note:*** Aliquot 15 μL/tube in 0.5 mL tubes. Store at −20°C for up to 12 months.

### SB431542 stock (15 mM)

Dilute 5 mg SB431542 in 866.5 μL of DMSO.

***Note:*** Aliquot 6.7 μL/tube in 0.5 mL tubes. Store at −20°C for up to 12 months.

### Acetic acid stock (10 mM)

Dilute 2 μL 17.4 M acetic acid in 3.478 mL H2O.

***Note:*** Filter the acetic acid stock solution through a 0.2 μm filter. Store at 4°C for up to 3 months.

### hLIF stock (100 μg/mL)

Dilute 25 μg hLIF in 250 μL 10 mM acetic acid.

***Note:*** Aliquot 5 μL/tube in 0.5 mL tubes. Store at −20°C for up to 6 months.

### N-2 supplement (100×)

***Note:*** Aliquot 0.5 mL/tube in 0.5 mL tubes. Store at −20°C for up to 12 months.

### B-27 with vitamin A supplement (50×)

***Note:*** Aliquot 1 mL/tube in 1.5 mL tubes. Store at −20°C for up to 6 months.

### StemMACS Y-27632 stock (10 mM)

Dilute 2 mg Y27632 in 624 μL of DMSO.

***Note:*** Aliquot 50 μL/tube in 0.5 mL tubes. Store at −20°C for up to 12 months.

### GFR Matrigel stock

***Note:*** To aliquot matrigel use cold P1000 tips. Put sterile tip box in −80 freezer a couple of hours before aliquoting. Aliquot 0.9 mL/tube in 1.5 mL tubes. Store at −20°C for up to 6 months.

#### Dithiothreitol solution (100 mM)

**CRITICAL:** Make fresh the day of use. Solution can be stored at −20°C and be used later the same day.

Weigh 15.4 mg of DTT. Add deionized water to bring the total volume to 1 mL. Mix well until the DTT is fully solubilized.

#### Iodoacetamide solution (550 mM)

**CRITICAL:** Make fresh the day of use as IAM is light-sensitive and degrades over time. If storing, keep the solution in a dark container at 4°C and use within a few hours for optimal activity.

Weigh 101.7 mg of IAM. Add deionized water to bring the total volume to 1 mL. Mix well until the IAM is fully solubilized.

### Ammonium bicarbonate solution (1 M)

Weigh 790.6 mg of ABC. Add deionized water to dissolve the ammonium bicarbonate, then adjust the final volume to 10 mL. Measure the pH with a pH meter. Ammonium bicarbonate typically yields a solution with a pH close to 8.0. If needed, adjust the pH very slightly using ammonium hydroxide (to raise the pH) or hydrochloric acid (to lower it).

#### Urea (8 M) in 100 mM ABC

**CRITICAL:** Make fresh the day of use.

Weigh 4.8 g urea. Add 1 mL of 1 M ABC pH 8.0. Add deionized water to make 10 mL. Stir well until urea is completely dissolved.

#### DTT (10 mM) and urea (8 M) in 0.1 M ammonium bicarbonate

Weigh 4.8 g urea. Add 1 mL of 1 M ABC solution. Add 1 mL of 100 mM DTT. Add deionized water to bring the total volume to 10 mL. Gently swirl or stir the solution to ensure it is well mixed.

#### Iodoacetamide (55 mM), urea (8M), and ammonium bicarbonate (0.1 M)

Weigh 4.8 g urea. Add 1 mL of 1 M ABC stock. Add 1 mL of 550 mM IAM stock. Add deionized water to bring the total volume to 10 mL. Gently swirl or stir the solution to ensure it is well mixed.

#### Ammonium bicarbonate (50 mM)

Pipette 500 μL of 1 M ABC. Add 9.5 mL of deionized water. Gently swirl or stir the solution to ensure it is well mixed.

**Table:** Metabolite extraction solution

Methanol	Add 500 mL to 1 L glass graduated cylinder.
Acetonitrile	Add 300 mL.
Water	Add 200 mL.

***Note:*** Pour into clean glass bottle and store capped at −20°C up to 6 months. When time-restricted, solution may be cooled in −80°C freezer for 30 min prior to use.

**Table:** 4% SDS in 100 mM triethylammonium bicarbonate pH 7.0

4% SDS in 100 mM triethylammonium bicarbonate (TEAB) pH 7.0	
Sodium dodecyl sulfate (SDS)	Weigh 0.4 g SDS.
TEAB (1 M)	Add 1 mL of 1 M TEAB pH 7.0.
Water	Add deionized water to a total volume of 10 mL.

***Note:*** Store at room temperature for short-term use (up to 1 week).

#### TEAB (100 mM) in 90% methanol

Add 5 mL of TEAB (1 M) to a 50 mL Falcon conical centrifuge tube. Add 45 mL methanol. Swirl or stir to mix.

***Note:*** Store at 4°C for up to a month.

#### TEAB stock (50 mM)

Add 2.5 mL of TEAB (1 M) to a 50 mL Falcon conical centrifuge tube. Add 47.5 mL of deionized water. Swirl or stir to mix.

***Note:*** Store at 4°C for up to 3 months.

## Step-by-step method details

### Preparation of neuronal induction medium

**Timing: 30 min**

This step outlines the preparation of the NIM medium, which supports the induction and maintenance of iNSCs. It details the precise composition and preparation steps required to ensure medium consistency and sterility, which are critical for reproducible neuronal differentiation and downstream metabolic analyses.

1. Preparation of 50 mL NIM.
  a. Mix the components of NIM: 24mL DMEM/F12, 24 mL Neurobasal medium, 1 mL B27 (end concentration 1×), 0.5 mL N2 (end concentration 1×), 0.5 mL glutamax (end concentration 1%), 15 μL CHIR99021 (end concentration 3 μM), 5 μL hLIF (end concentration 10 mg/mL), 6.7 μL SB-431542 (end concentration 2 μM).

***Note:*** Small molecules (CHIR99021, hLIF, SB-431542) must be added fresh to the NIM before use.

For more details on NIM preparation and iNSC maintenance, please refer to Ionescu et al. (2024).^1^ For metabolic analysis in the same set of samples, the same batch of medium should be used. All media and supplements were sterile-filtered through a 0.22 μm filter under aseptic conditions in a biosafety cabinet prior to use.

### Preparation of Matrigel-coated plates

**Timing: 30 min**

This step describes the preparation of Matrigel-coated culture plates, which provide an extracellular matrix environment essential for the adherence and maintenance of iNSCs. It includes detailed steps for thawing, diluting, and coating with Matrigel to ensure consistent surface coverage and optimal cell attachment, as well as storage and handling considerations to preserve coating integrity.

2. Thawing, diluting and coating using Matrigel.
  ***Note:*** Pre-chill DMEM/F12 at 4°C for at least 1 h.
  a. Thaw one aliquot of Matrigel slowly (stored at −20°C) by placing it in 4°C fridge.
  b. Dilute Matrigel aliquot 1:20 with appropriate amount of DMEM/F12 (18 mL).
    ***Note:*** The final Matrigel concentration varies slightly between lots, as the protein content is lot-specific, but a 1:20 dilution typically yields a working concentration of approximately 0.4–0.6 mg/mL.
  c. Mix well by gently inverting the tube.
  d. Pipette 0.5 mL of diluted Matrigel into each well of a 12-well plate, ensuring even cover of the well surface.
    **CRITICAL:** Work quickly since Matrigel polymerizes at room temperature.
    ***Note:*** Use 1 mL/well diluted Matrigel for 6-well plates and 0.2 mL/well diluted Matrigel for 24-well plates.
  e. Label plates with date and seal all plates well with parafilm.
  f. Incubate in fridge at 4°C overnight.
  g. On the next day, Matrigel can be removed and replaced by the same amount of DMEM/F12. Keep Matrigel dilution in order to re-use (Matrigel may be used twice).
    ***Note:*** Matrigel plates can be stored for up to 1 week in the fridge. Do not use longer!
    **Pause point:** Coated plates can be wrapped with parafilm and stored at 4°C for up to 1 week.
    ***Note:*** Medium remaining in the coated plate will continuously evaporate even at 4°C. Avoid using the plate if part of the culturing surface has dried.

### Recovery of iNSCs from frozen stocks

**Timing: 20 min**

This step outlines the procedure for recovering iNSCs from frozen stocks to ensure optimal post-thaw viability and attachment. It details each step from thawing and washing to seeding on Matrigel-coated plates in NIM, including the use of ROCK inhibitor to enhance cell survival during recovery.

3. Recovery of iNSCs.
  a. Thaw cells in a cryovial by placing it in a water bath (37°C).
  b. When there is only a small ice crystal floating, place cryovial in a biosafety cabinet and add 1 mL of fresh DMEM/F12 to the cells.
  c. Place cell suspension into a 15 mL Falcon tube containing 8 mL DMEM/F12.
  d. Pellet cells by centrifugation at 300 × *g* for 5 min.
  e. Carefully remove supernatant and resuspend pellet in an appropriate amount of NIM medium (depending on cell culture plate format – 1.5 mL/well NIM for 6-well plates; 0.75 mL/well NIM for 12-well plates, 0.5 mL/well NIM for 24-well plates).
    ***Note:*** In general, always thaw the cells in the same plate format they have been frozen (e.g., if 1 well of a 6-well plate has been cryopreserved, thaw cells again in 1 well of a 6-well plate!)
  f. Add ROCK inhibitor Y-27632 (end concentration 10 μM).
  g. Seed cells in GFR-matrigel coated cell culture vessels.
  h. On the next day, refresh medium to remove ROCK inhibitor by aspirating the culture medium from the cell culture vessels and replacing it with fresh NIM medium.

### Maintenance and passaging of iNSCs

**Timing: 20 min for step 5**

**Timing: 30 min for step 6**

This step describes the routine maintenance and passaging of iNSCs to preserve their viability, morphology, and multipotent state. It details the optimal timing and procedure for medium exchange and enzymatic dissociation using Accutase, followed by reseeding onto Matrigel-coated plates in NIM with ROCK inhibitor to support healthy proliferation and stable long-term culture.

4. Medium exchange.
  a. Change the media every 2^nd^-3^rd^ day or when yellowing of the media is present. For one well of a 6-well plate, aspirate spent medium and add 1.5 mL fresh NIM medium.

**CRITICAL:** Take NIM out of the 4°C fridge and keep at room temperature for 1 h prior to medium exchange.

5. Splitting of iNSCs
  ***Note:*** Ideally, cells should be split when they are reaching 50–70% confluency. In this protocol a 6-well plate was used as starting material. See below for volumes of Accutase and media used if another vessel format is used.
  a. Remove medium.
  b. Add 500 μL Accutase to each well, mix gently by moving the plate.
    **CRITICAL:** Do not prewarm the Accutase.
  c. Incubate the plate at 37°C for 6–10 min, check detachment of the cells under the microscope.
  d. Stop the reaction of the Accutase by adding 1 mL DMEM/F12 to the well. Detach remaining cells from the bottom of the plate by pipetting gently against the well.
    ***Note:*** Depending on the cell culture plate format a different volume of Accutase may be used – 0.25 mL/well Accutase for 12-well plates, 0.15 mL/well Accutase for 24-well plates.
    ***Note:*** The DMEM/F12 should be pre-warmed to 37°C to maintain cell viability and prevent thermal shock during dissociation.
  e. Transfer the resuspended cells into a 15 mL Falcon tube.
  f. Use another 1 mL of DMEM/F12 to detach remaining cells, resuspend, and transfer into Falcon tube. Repeat this step.
  g. Pellet cells by centrifugation at 300 × *g* for 5 min.
  h. Carefully remove supernatant.
  i. Resuspend pellet in 1 mL of NIM medium.
  j. Remove GFR-matrigel from a new 6-well plate and place 1.5 mL NIM medium into each well.
    ***Note:*** Depending on the cell culture plate format a different volume of NIM may be used – 0.75 mL/well NIM for 12-well plates, 0.5 mL/well NIM for 24-well plates.
  k. Seed cells in desired dilution (e.g., 1:10, 1:20, 1:30) by pipetting appropriate volume of cell suspension into a new well. Add ROCK inhibitor 1:1000 (10 μM).
  l. Allow the cells to grow in a humified incubator, 5% CO_2_, 37°C.

### Cryopreservation of iNSCs

**Timing: 30 min**

This step outlines the procedure for cryopreserving induced neural stem cells (iNSCs) to ensure long-term storage and recovery without loss of viability or phenotype. It details the preparation of freezing medium, handling of cells during freezing, and the controlled-rate cooling process prior to transfer to liquid nitrogen for stable preservation.

6. Freezing iNSCs.
  a. Once 50–70% confluent, split the cells as described above until Step 6h.
  b. Resuspend cells in freezing medium (90% KnockOut serum replacement, 10% DMSO).
    ***Note:*** The freezing medium should be kept cold (on ice or at 4°C) during preparation and cell resuspension to minimize DMSO toxicity and preserve cell viability.
  c. Place cell suspension in cryovials, label, and place vials into a Mr. Frosty.
  d. Place the Mr. Frosty and the cells at −80°C overnight.
  e. Place cells in liquid N_2_ the following day.

### iNSC preparation for isotopic tracing

This step describes how to prepare iNSCs for stable isotope tracing experiments to analyze metabolic fluxes. It outlines the seeding strategy, culture conditions, and tracer administration using [^13^C_6_]-glucose and [^13^C_5_, ^15^N_2_]-glutamine, ensuring reproducible experimental setup and accurate normalization for downstream UHPLC-MS–based metabolomics.

#### Day 0

**Timing: 30 min**

7. Seed enough cells into 12-well plates for UHPLC-MS analysis as described above (step 6).
  a. Prepare 3 wells of cells for each treatment, which will be collected individually as three replicates.
  b. Additionally, plate 3 wells with only culture media to serve as a baseline of media without cells. Multiple plates will be needed.

***Note:*** In our experience, a sufficient number of iNSCs required for high quality metabolomics analysis is 200,000. When iNSCs reach 70% confluence, the cell number in each well of a 12-well plate is over 200,000. This number may vary for different cell lines. It is important to avoid over-confluence of the culture, therefore cells need to be monitored under the microscope daily. Additionally, other cell characteristics such as cytoplasmic abundance may influence the number of cells needed to obtain a reliable signal.

**CRITICAL:** Accurate cell counts are important for normalization. Prior to metabolomics data acquisition, multiple rounds of experiments are often conducted to evaluate the impact of specific treatment on cell proliferation. As an alternative approach for normalization, cellular protein concentration from each well may also be used. However, if albumin or FBS is used in the culture medium, it is important to make sure that exogenous proteins are rinsed off effectively before protein measurement.

#### Day 1

**Timing: 10 min**

8. Refresh medium. For 12-well plates, add 0.75 mL fresh NIM medium per well. Add appropriate treatments.

***Note:*** Treatment period is flexible depending on the experiment design. Medium should be changed every 2–3 days until the day of metabolic tracing. In our experiments, tracing is performed on day 2, i.e., 24 h after the final medium change.

**CRITICAL:** Avoid over-confluence of cells.

#### Day 2

**Timing: 10 min**

9. Run glucose tracer experiments on day 2 after seeding.
  a. Remove the culture medium.
  b. Add glucose-free NIM supplemented with D-Glucose-^13^C_6_ (Thermo Fisher, 20.4 mM).

***Note:*** Glucose-free NIM comprises of DMEM/F12 without glucose and Neurobasal (1:1), supplemented with N-2 supplement (1×, Thermo Fisher), Glutamax (Thermo Fisher, 3.2 mM), pyruvate (Agilent, 0.35 mM), B-27 supplement (1×, Thermo Fisher), CHIR99021 (3 mM, Cell Guidance Systems), SB-431542 (2 mM, Cayman Chemicals) and hLIF (10 ng/mL, Cell Signaling Technology).

***Note:*** Collected samples after 30 min (for optimal resolution of glycolysis), 6 h (for assessment of protein acetylation and for optimal resolution into TCA cycle) and 24 h (for optimal resolution into TCA cycle and anabolic metabolic pathways).

10. Run glutamine tracer experiment on day 2 after seeding.
  a. Remove the culture medium.
  b. Add glutamine-free NIM supplemented with L-Glutamine-^13^C_5_, ^15^N_2_ (Thermo Fisher, 3.2 mM).

***Note:*** Glutamine-free NIM comprises of DMEM/F12 without glutamine and Neurobasal (1:1), supplemented with N-2 supplement (1×, Thermo Fisher), glucose (Agilent, 20.4 mM), pyruvate (Agilent, 0.35 mM), B-27 supplement (1×, Thermo Fisher), CHIR99021 (3 mM, Cell Guidance Systems), SB-431542 (2 mM, Cayman Chemicals) and hLIF (10 ng/mL, Cell Signaling Technology).

***Note:*** Collected samples after 6 h and 24 h (for optimal resolution into TCA cycle and anabolic metabolic pathways).

### Medium and cell collection following isotopic tracing

**Timing: 1 h**

This step describes how to collect conditioned medium and cells following isotopic tracing experiments for downstream metabolomic analysis. It provides detailed guidance on harvesting and processing samples under cold conditions to preserve metabolite integrity, including removal of debris, normalization by cell count, and rapid snap-freezing to facilitate the generation of high-quality UHPLC-MS data.

11. Collect conditioned medium.
  a. Transfer the culture medium from each well to a 1.5-mL Eppendorf tube. Spin at 300 × *g* for 5 min to remove cellular debris
  b. Transfer 100 μL of the supernatant into a new 0.2-mL Eppendorf tube for UHPLC-MS analysis and place on dry ice. Freeze samples at −80°C until ready for extraction.
12. Collect cells.
  a. Add 250 μL Accutase to each well, mix gently by moving the plate.
  b. Incubate the cells at 37°C for 6–10 min, check detachment of the cells under the microscope.
  c. Once the cells have started detaching, add 0.5 mL of cold PBS (0°C–4°C) to the well. Detach remaining cells from the bottom of the plate by gently pipetting against the well.
    ***Note:*** Depending on the cell culture plate format a different volume of Accutase may be used – 0.5 mL/well Accutase for 6-well plates, 0.15 mL/well Accutase for 24-well plates.
    ***Note:*** PBS should be pre-cooled in the fridge. An appropriate volume should be transferred into a smaller vessel and kept on ice throughout the sample collection.
  d. Transfer the resuspended cells into a 1.5-mL Eppendorf tube kept on ice.
  e. Use another 0.75 mL of cold PBS to detach remaining cells, resuspend them and transfer into Eppendorf tube kept on ice.
  f. Pellet cells by centrifugation at 300 × *g* for 5 min at 4°C.
  g. Carefully remove supernatant.
  h. Resuspend pellet in 1 mL of cold PBS medium for cell counting. Determine the live cell count in each sample using a hemocytometer or automated cell counter.
    ***Note:*** Make a note of the cell counts in each sample. Samples for metabolomics are input-normalized prior to data acquisition.
  i. Pellet cells once more by centrifugation at 300 × *g* for 5 min at 4°C.
  j. Carefully aspirate supernatant.
    **CRITICAL:** Salts from PBS and other wash buffers adversely affect metabolite ionization. To ensure good quality omics data, make sure to remove supernatant as best as possible without aspirating the cell pellet. This can be facilitated by tilting the tube and applying a gentle jerking motion to move remaining droplets to the side walls of the tube, where they can be safely aspirated. Please note that at the cell counts used in the present experiment, the cell pellets are difficult to visualize.
  k. Snap freeze the cell pellets on dry ice. Store samples at −80°C until ready for extraction.

**Table 1:** LC settings for metabolomics

Liquid chromatograph	Vanquish Horizon
Column type	Acquity BEH C18 (Waters)
Column dimensions	2.1 x 100 mm
Column particle size	1.7 μm
Autosampler temperature	7°C
Column temperature	45°C
Injection volume - cells	10 μL
Injection volume - cell supernatants	10–20 μL
Mobile phase A - positive ion mode	water with 0.1% formic acid
Mobile phase B - positive ion mode	acetonitrile with 0.1% formic acid
Mobile phase A - negative ion mode	10 mM ammonium acetate
Mobile phase B - negtive ion mode	10 mM ammonium acetate in 1:1 acetonitrile:methanol

**Table 2:** Metabolomics gradient

Time (min)	% B
0	5
0.5	5
1.1	95
2.75	95
3	5
5	5

**Table 3:** MS settings for metabolomics

Mass spectrometer	Thermo Orbitrap Exploris 120
Ionization source	HESI
Gas type	Nitrogen
Sheath gas	50 arb
Auxiliary gas	10 arb
Sweep gas	1 arb
Spray voltage - positive ion mode	3.4 kV
Spray voltage - negative ion mode	3.0 kV
Ion transfer tube temperature	320°C
Vaporizer temperature	350°C
MS mode	MS^1^
Scan range	65–975 m/z
Resolution	120,000
RF lens	70%

**Table 4:** LC settings for lipidomics

Liquid chromatograph	Vanquish Horizon
Column type	Kinetex C18 (Phenomenex)
Column dimensions	2.1 x 30 mm
Column particle size	1.7 μm
Autosampler temperature	7°C
Column temperature	50°C
Injection volume - cells	10 μL
Injection volume - cell supernatants	10–20 μL
Mobile phase A	5 mM ammonium acetate in 3:1 water:acetonitrile
Mobile phase B	5 mM ammonium acetate in 9:1 isopropanol:acetonitrile

**Table 5:** Lipidomics gradients

Negative ion mode			Positive ion mode		
Time (min)	% B	Flow rate (mL/min)	Time (min)	% B	Flow rate (mL/min)
0	10	0.3	0	30	0.3
3	95	0.3	3	100	0.3
4.2	95	0.3	4.2	100	0.3
4.3	10	0.45	4.3	30	0.4
4.9	10	0.4	4.9	30	0.4
5	10	0.3	5	30	0.3

**Table 6:** MS settings for lipidomics

Mass spectrometer	Thermo Q Exactive
Ionization source	HESI
Gas type	Nitrogen
Sheath gas	45 arb
Auxiliary gas - positive ion mode	25 arb
Auxiliary gas - negative ion mode	10 arb
Sweep gas	0 arb
Spray voltage - positive ion mode	4.0 kV
Spray voltage - negative ion mode	3.0 kV
Capillary temperature	320°C
RF lens	50%
MS mode	ddMS^2^
Scan range for MS^1^ - positive ion mode	125–1500 m/z
Scan range for MS^1^ - negative ion mode	150–1500 m/z
Resolution - MS^1^	70,000
Cycle number (top N)	10
Resolution - MS^2^	17,500
Dynamic exclusion	8 s
Stepped normalized collision energy - positive ion mode	25%, 35%
Stepped normalized collision energy - negative ion mode	25%, 40%

**Table 7:** LC and gradient settings for proteomics

Liquid chromatograph	Evosep one
Column type	Pepsep
Column dimensions	150 μm x 15 cm
Resin type	ReproSil C18
Resin particle size	1.9 μm, 120 A
Column temperature	40°C
Injection amount	200 ng of peptides
Mobile phase A	Water with 0.1% formic acid
Mobile phase B	Acetonitrile with 0.1% formic acid
Gradient	88 min (Bruker program)
Flow rate	250 nL/min

**Table 8:** MS parameter settings for proteomics

Mass spectrometer	Bruker timsTOF
Ionization source	Nano-electrospray via CaptiveSpray
Gas type	Nitrogen
Dry gas	3 L/min
Spray voltage	1.5 kV
Dry temperature	200°C
MS mode	PASEF
Ramp time	100 ms
Accumulation time	100 ms
Scan range for MS^1^ and MS^2^	100–1700 m/z
Ion mobility scan range	0.7–1.5 Vs/cm^2^
Isolation window for precursor selection	±1 Th
Minimum intensity for precursor selection	500 counts
Collision energy (ion mobility-dependent)	20–59 eV
Precursors with repeated analysis	500–20,000 counts
Ions dynamically excluded	>20,000 counts
Dynamic exclusion	0.4 min
Cycle number (top N)	10

**Figure 2:**
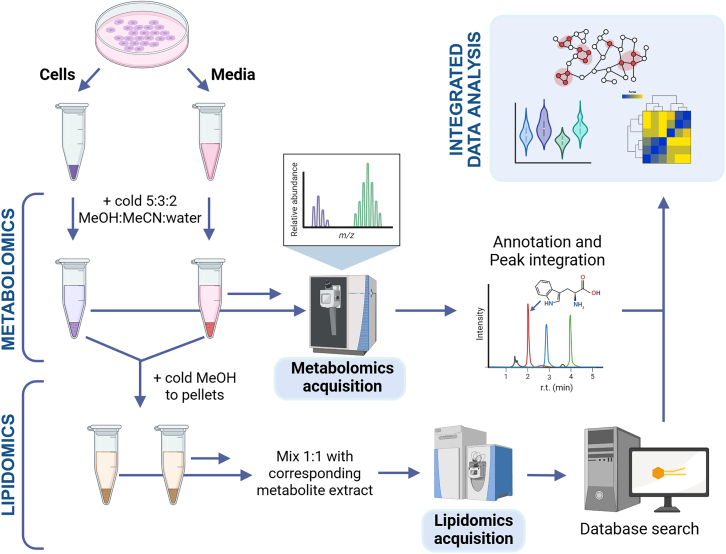
Sample preparation and data acquisition for metabolomics and/or lipidomics on single specimens of cells and/or cell supernatants

**Figure 3:**
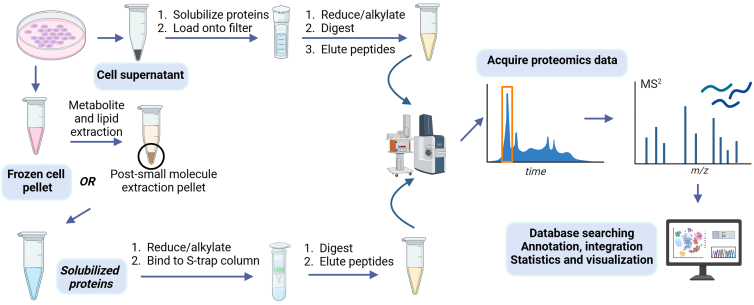
Sample preparation and data acquisition for proteomics subsequent to metabolomics and/or lipidomics using the same specimens of cells and/or cell supernatants

### Metabolite extraction for cells and cell supernatants

**Timing: 1 h or more depending on the number of samples**

13. Prepare for metabolite extraction.
  a. Obtain tubes containing frozen cell pellets and store on the bench in a box containing dry ice. Obtain tubes containing frozen cell supernatant (i.e., conditioned media) aliquots and thaw on ice.
  b. For cell supernatant tubes, transfer 20 μL of specimen to a new 1.7–2.0 mL microcentrifuge tube on ice.
  c. Remove metabolite extraction solution (MES) from the −20°C freezer and store on the bench in a box on ice.
14. Extract metabolites.
  a. Add MES to each sample using a micropipette (Figure 2).
    ***Note:*** For cell pellet tubes, add MES to a concentration of 2 million cells per mL. For cell supernatant tubes, add 480 μL of MES. If the same cell pellet specimens will be used for subsequent lipidomics extraction, it may be desirable to use a concentration of 4 million cells per mL for metabolomics extraction.
    ***Note:*** As MES is 80% organic and low viscosity, dripping from the pipette tip may occur. Transfer quickly and efficiently then close the tube cap.
    ***Note:*** Two million cells per mL is a rule of thumb for an average size eukaryotic cell. The extraction concentration may be adjusted according to cell type and size. For example, it is desirable to extract metabolites from stem cells at a higher concentration.
    ***Note:*** If accurate cell counts are not available, add equal amounts of MES to each sample and plan to normalize samples or data to protein concentration.
    ***Note:*** Volumes for cell supernatant extraction can be scaled as needed while maintaining 1:25 ratio.
  b. Transfer tubes to a vortex with foam accessory to hold microcentrifuge tubes. Vortex samples vigorously for 30 min at 4°C such as in a cold room or refrigerator.
  c. Pellet insoluble material via centrifugation for 10 min, 4°C, 14,000 g.
    **CRITICAL:** Speeds at or above 10,000 g are sufficient. Refrigerated centrifugation is essential.
15. Prepare quality control (QC) pooled sample.
  a. Transfer 5 mL of clarified supernatant from each cell extract tube to a single autosampler vial contained in a box on ice. Gently pipette to mix.
  b. Repeat this process for extracts from cell supernatants to yield a second QC vial.

***Note:*** Volume can be increased or decreased as needed to cover 2 injections at the beginning of the sequence, 1 at the end, and injections every 5–15 samples throughout the sequence. Two polarities will be used, so number of injections of QC vial should be doubled. If insufficient volume exists in the extracts due to low cell counts, a pool of cell supernatant extracts can be used for the cell extract sequence.

16. Prepare autosampler vials containing sample extracts.
  a. Add 50 μL of clarified supernatant (Figure 1) from each cell extract tube to a labeled autosampler vial contained in a box on ice.

**CRITICAL:** Cap vials immediately after transfer. Inspect to ensure the absence of an air bubble below the solution. If an air bubble is apparent, a hard shake downward will displace it.

***Note:*** If a concentration of 4e6 cells/mL was used in Step 14a, dilute extracts with 50 μL of cold MES to obtain a concentration of 2e6 cells/mL. Maintain vials in a box on ice during transfers.

17. Store unused sample extracts.
  a. Move unused metabolite extract tubes to −80°C freezer for short or long term storage.

***Note:*** If protein quantification or proteomics are planned, first remove all remaining supernatant and transfer to a new tube. Then resuspend insoluble pellet in buffer such as PBS or urea for desired downstream protein quantification assay.

***Note:*** If −80°C-stored tubes are needed at a later date for analysis, thaw on ice, vortex to mix material, then spin as in Step 14c.

18. Prepare instrument sequence.
  a. Randomize the order of sample runs and include blank and QC injections as appropriate.

***Note:*** We recommend randomizing the cell extracts and supernatant extracts separately and for each sample type to have its own QC vial.

***Note:*** We run samples in one polarity followed by the other. It is also possible to use polarity switching methods to reduce instrument time by half. Testing and optimization should be performed in advance to ensure sufficient data points across each peak.

19. Separate and quantify metabolites in the supernatant-containing autosampler vials using ultra-high-pressure liquid chromatography coupled to mass spectrometry.

***Note:*** Our platform utilizes Thermo Vanquish UHPLCs coupled to Thermo Q Exactive or Thermo Orbitrap Exploris mass spectrometers. Eluate is introduced to the mass spectrometer via electrospray ionization.

### Lipid extraction for cells and cell supernatants following metabolomics processing

**Timing: 1 h or more depending on the number of samples**

20. Prepare for lipid extraction.
  a. Obtain insoluble material resulting from metabolomics extraction (Figure 2) and ensure that supernatant has been transferred to new tube (Step 17).
  b. Obtain high quality cold methanol and place in a box with ice.
21. Extract lipids from cell and cell supernatant tubes.
  a. Add cold high-quality methanol to pelleted material at a concentration of 4e6 cells/mL based on original cell counts.
    ***Note:*** Methanol is non-viscous and will drip from the pipette tip. Transfer efficiently.
  b. Briefly vortex on benchtop to resuspend solid material then store without agitation at −20°C for 30 min.
  c. Vortex vigorously for 30 min at 4°C.
  d. Pellet insoluble material by centrifugation 18,213 × *g*, 10 min, 4°C.
    **CRITICAL:** Speeds at or above 10,000 g are sufficient. Refrigerated centrifugation is essential.
22. Prepare autosampler vials for lipidomics.
  a. Mix 50 μL of clarified supernatant from each tube with 50 μL of metabolite extract from Step 16 in an autosampler vial. Ensure mixing via vortex or pipette.
  b. Remove 5–10 μL from the autosampler vial and place into a QC autosampler vial. The QC vial will contain 5–10 μL of each extract pooled into a single vial.

**CRITICAL:** Cap vials immediately after transfer. Maintain vials in a box on ice during transfers.

23. Prepare and execute lipidomics instrument sequence.
  a. Randomize the order of sample runs and include blank and QC injections as appropriate.
  b. Separate and quantify lipids in the autosampler vials using ultra-high-pressure liquid chromatography coupled to mass spectrometry.

***Note:*** Our platform utilizes Thermo Vanquish UHPLCs coupled to Thermo Q Exactive mass spectrometers. Eluate is introduced to the mass spectrometer via electrospray ionization.

24. Store remaining lipid extracts.
  a. Remove remaining lipid extracts (i.e., supernatant) from tubes described in step 22a, transfer to a new tube, and store at −80°C.

***Note:*** Tubes containing the pellet will be used for proteomics analysis.

### Lipid extraction for cells and cell supernatants without upstream metabolomics

**Timing: 1 h or more depending on the number of samples**

25. Prepare for lipid extraction.
  a. Obtain tubes containing frozen cell pellets and store on the bench in a box containing dry ice.
  b. Obtain tubes containing frozen cell supernatant (i.e., conditioned media) aliquots and thaw on ice.
  c. Obtain cold high-quality methanol in a sufficient volume to extract lipids at 2 million cells per mL.

***Note:*** We recommend high purity methanol suitable for LC-MS applications.

26. Extract lipids.
  a. Add cold methanol to each cell pellet tube via micropipette to a concentration of 2 million cells per mL.
    ***Note:*** Methanol is non-viscous and will drip from the pipette tip. Transfer efficiently.
    ***Note:*** Two million cells per mL is a rule of thumb for an average size eukaryotic cell. The extraction concentration may be adjusted according to cell type and size. For example, it is desirable to extract lipids from stem cells at a higher concentration.
    ***Note:*** If accurate cell counts are not available, add equal amounts of methanol to each sample and plan to normalize samples or data to protein concentration.
  b. Vortex cell supernatant tubes briefly to homogenize, then transfer a 10 μL aliquot of cell supernatant to a new 1.5–2.0 mL microcentrifuge tube on ice, then add 90 μL of cold high-quality methanol.
  c. Vortex suspensions briefly then store without agitation at −20°C for 30 min.
  d. Vortex vigorously for 30 min at 4°C.
  e. Pellet insoluble material by centrifugation 18,213 × *g*, 10 min, 4°C.
    **CRITICAL:** Speeds at or above 10,000 g are sufficient. Refrigerated centrifugation is essential.
27. Prepare QC vial and autosampler vials of extracts.
  a. Follow procedures described in steps 15–17 to aliquot lipidomics extracts into autosampler vials and prepare a QC vial.
28. Store unused extracts.
  a. Remove remaining supernatant from tubes described in step 27 via micropipette and transfer to a new tube.

***Note:*** Tubes containing the pellet will be used for proteomics analysis.

29. Acquire lipidomics data.
  a. Separate and quantify lipids in the supernatants using ultra-high-pressure liquid chromatography coupled to mass spectrometry.

***Note:*** Our platform utilizes Thermo Vanquish UHPLCs coupled to Thermo Q Exactive mass spectrometers. Eluate is introduced to the mass spectrometer via electrospray ionization.

### Sample processing for proteomics—Cells

**Timing: Hands-on time, ∼3–4 h; digestion, 6 h**

30. Resuspend pelleted material yielded by metabolite or lipid extractions (Figure 2) to solubilize proteins in preparation for proteomics characterization (Figure 3).
  a. Solubilize pelleted material by adding 4% SDS in 100 mM triethylammonium bicarbonate (TEAB) using the volumes that match the amount of MES added for metabolite extraction or methanol added for lipid extraction.
  b. Vortex for 15 min at ambient temperature.
  c. Spin briefly to ensure all liquid is at the bottom of the tubes.
31. Reduce and alkylate protein cysteines.
  a. Label 1.5 mL conical-bottom Eppendorf tube and transfer a 50 μL aliquot of sample.
  b. Add DTT to a final concentration of 10 mM.
  c. Vortex for 5 s, then spin down to ensure all liquid is at the bottom of the tubes.
  d. Place the tubes into the heating block at 55°C for 30 min.
  e. Remove tubes from the heating block and allow them cool to ambient temperature on the benchtop.
  f. Add IAM to a final concentration of 25 mM.
  g. Vortex for 5 s, then spin down to ensure all liquid is at the bottom of the tubes.
  h. Incubate in the dark for 30 min.
32. Bind protein solution to column for digestion.
  a. Add phosphoric acid to a final concentration of 1.2% (v/v).
  b. Add six volumes of binding buffer (90% methanol; 100 mM TEAB pH 7.0). Mix gently.
  c. Load resulting samples onto S-Trap micro spin columns.
    **CRITICAL:** Using the correct acid concentration (1.2%) and binding buffer volume (6× sample volume**)** ensures uniform protein precipitation across samples, which is critical for reproducible peptide yields and quantitative LC–MS results.
  d. Centrifuge at 1500 × *g* for 2 min to allow binding.
  e. Collect the flow-through and reload onto the S-Trap micro spin column. Repeat this step twice more for a total of 3 times.
    ***Note:*** For best results, rotate the S-Trap micro spin column 180° between the centrifugations of binding and wash steps. This is especially important when using a fixed-angle rotor because the spin column does not experience homogenous flow.
  f. Add 300 μL of binding buffer.
  g. Centrifuge at 1500 × *g* for 2 min. Repeat 3 times and discard flow through. After each centrifugation step, ensure that all solution has passed through the S-Trap column. Centrifuge longer if needed.
    ***Note:*** Visually confirm all sample has passed through the column; if not, centrifuge again until all sample has passed through.
33. Digest proteins.
  a. Transfer S-Trap micro column to a clean 2 mL sample tube for digestion.
  b. Add 1 μg of sequencing-grade trypsin (Promega) dissolved in 125 μL of 50 mM TEAB onto the S-Trap micro spin column.
    **CRITICAL:** Ensure there is no air bubble between the digestion buffer and the S-trap micro spin matrix.
    ***Note:*** Other proteases, e.g. Lys-C, may be used in place of trypsin depending on protein sequences of interest.
  c. Incubate at 37°C for 6 h.
    ***Note:*** Proper time and temperature optimize digestion completeness while avoiding overdigestion. Critical for reproducible, high-quality LC–MS results.
34. Elute peptides from S-Trap column.
  ***Note:*** Efficient elution recovers as many peptides as possible. Skipping or incomplete elution can lead to peptide loss and reduce proteome coverage.
  a. Add 100 μL of 50 mM TEAB then spin 1500 × *g* for 2 min.
  b. Add 100 μL of 0.2% formic acid in water then spin 1500 × *g* for 2 min.
  c. Add 100 μL of 80% acetonitrile in 0.1% FA then spin 1500 × *g* for 2 min.
  d. Combine the peptide solutions.
  e. Evaporate the solvent using a SpeedVac.
  f. Reconstitute the residue in 100 μL of 0.1% FA.
35. Store the samples at −80°C for long-term storage or proceed directly to mass spectrometry analysis.

***Note:*** Freezing at −80°C prevents proteolytic degradation and chemical modifications (oxidation, deamidation) that can occur at higher temperatures.

### Sample processing for proteomics—Conditioned media/cell supernatants

**Timing: Hands-on time, ∼3–4 h; digestion, 12–16 h**

36. Load sample onto Vivacon spin filter (Figure 3).
  a. Mix 100 μL of conditioned media with 100 μL of 8 M urea, 0.1 M ABC, pH 8.0 on the Vivacon spin filter.
  b. Centrifuge at 14,000 g for 15 min and discard flow through from the collection tube.
37. Denature proteins then reduce and alkylate cysteines.
  a. Add 100 μL of 8 M urea, 0.1 M ABC to the Vivacon spin filter, centrifuge at 14,000 × *g* for 15 min and discard flow through from the collection tube.
  b. Repeat Step 44 once more.
  c. Add 100 μL of 10 mM DTT in 8 M urea, 0.1 M ABC, incubate at room temperature for 20 min.
  d. Centrifuge at 14,000 g for 15 min and discard flow through.
    ***Note:*** Removes excess DTT that could interfere with alkylation.
  e. Add 100 μL of 55 mM IAM in 8 M urea, 0.1 M ABC, incubate 30 min in the dark.
  f. Centrifuge the Vivacon spin filter at 14,000 × *g* for 15 min. Discard flow through.
  g. Add 100 μL of 8 M urea, 0.1 M ABC to the Vivacon spin filter, centrifuge at 14,000 × *g* for 15 min and discard flow through from the collection tube
  h. Repeat step 44g twice.
  i. Add 100 μL of 50 mM ABC to the Vivacon spin filter, centrifuge at 14,000 × *g* for 15 min and discard flow through from the collection tube
    ***Note:*** This step replaces urea with MS-compatible buffer (ABC) to prepare proteins for enzymatic digestion.
  j. Repeat step 44i twice.
38. Digest proteins.
  a. Transfer the Vivacon spin filter to a new collection tube.
    **CRITICAL:** New collection tube prevents contamination from previous wash steps and ensures only clean, trapped proteins are digested.
  b. Digest proteins using sequencing grade modified trypsin (Promega) at 1/50 protease/protein (wt/wt) at 37°C overnight.
39. Recover peptides from the filter.
  a. Add 100 μL vol of 50 mM ABC to the Vivacon spin filter and centrifuge at 14,000 × *g* for 15 min.
  b. Repeat the elution once more to maximize recovery.
  c. Dry eluate using a SpeedVac.
  d. Reconstitute the residue in 100 μL of 0.1% FA for LC-MS analysis.

***Note:*** Sample pH can be confirmed to be between 2 to 3 with 1–2 μL sample using pH paper to ensure peptide stability, optimal binding to the C18 material, and consistent recovery.

40. Desalt peptide samples.
  a. Insert C18 spin tip into spin adapter seated in microcentrifuge tube.
  b. Wash C18 spin tip with 20 μL of Solvent B (0.1% FA in 80% acetonitrile) and centrifuge at 1000 × *g* for 60 s.
  c. Equilibrate the C18 spin tip with 20 μL of Solvent A (0.1% FA in water) and centrifuge at 1000 × *g* for 60 s.
  d. Transfer C18 spin tip and adapter to a new microcentrifuge tube.
  e. Pipette 20 μL of the sample onto the C18 spin tip and centrifuge at 1000 × *g* for 60 s. Reapply the sample to the C18 spin tip twice.
    ***Note:*** Multiple passes maximize peptide binding to the resin. Incomplete loading reduces recovery and may bias sample composition.
  f. Wash the tip by adding 20 μL of solvent A and centrifuging at 1000 × g for 60 s. Repeat three additional times.
  g. Transfer C18 spin tip and adapter to a new microcentrifuge tube.
  h. Elute the sample by adding 20 μL of solvent B and centrifuging at 1000 × g for 60 s. Repeat one additional time.
  i. Dry the eluate nearly to dryness using a SpeedVac, then re-suspend in 100 μL of 0.1% FA.
    **CRITICAL:** Avoid complete dryness, which can make peptides difficult to resuspend.
41. Prepare the Evotips following the vendor’s instructions.
  a. Wash dry Evotips with 20 μL of Solvent B (0.1% FA in 80% acetonitrile) and centrifuge at 800 × *g* for 60 s.
  b. Soak Evotips in 100 μL of 2-isopropanol until they turn pale white.
  c. Saturate the conditioned Evotips with 20 μL of Solvent A (0.1% FA in water) and centrifuge at 800 × *g* for 60 s
42. Load Evotips with peptide samples.
  a. Pipette 20 μL of samples into the Evotip.
  b. Centrifuge for 60 s at 800 × *g*.
  c. Wash the Evotip with 200 μL of Solvent A.
  d. Centrifuge for 60 s at 800 g. Repeat steps 42c-d 3 times.
    **CRITICAL:** Removes salts and other contaminants that can suppress ionization in LC–MS. Multiple washes ensure clean, reproducible sample loading.
  e. Add 200 μL of Solvent A to each Evotip and centrifuge briefly to keep tips wet.
  f. Add enough Solvent A to the bottom of the tray so the bottoms of the Evotips are submerged and store at 4°C with lid on until analysis.

### Metabolomics data acquisition parameters

**Timing: 3 h for the first 5 samples plus accompanying QC runs; add 1 h increments as needed per additional 4 samples and their accompanying QC runs**

43. Use liquid chromatography to separate metabolites prior to MS acquisition.
  ***Note:*** We utilize a Thermo Vanquish ultra high pressure liquid chromatograph (UHPLC) equipped with a Waters Acquity BEH C18 column (Table 1).
  a. Acquire data using a high throughput 5 min gradient in negative and positive ion modes – 2 runs per sample (Table 2).
    ***Note:*** We recommend running the full sample-randomized sequence in one polarity mode then again in the second polarity mode with several blank and QC injections between. The Vanquish UHPLC is coupled to a Thermo Q Exactive or Thermo Orbitrap Exploris mass spectrometer via a heated electrospray ionization (HESI) source (Table 3). Users of this protocol are encouraged to optimize analytical and startup parameters given their instruments and research questions to generate the best possible data.

### Lipidomics data acquisition parameters

**Timing: 3 h for the first 5 samples plus accompanying QC runs; add 1 h increments as needed per additional 4 samples and their accompanying QC runs**

44. Use liquid chromatography to separate lipids prior to MS acquisition.
  ***Note:*** We utilize a Thermo Vanquish ultra high pressure liquid chromatograph (UHPLC) equipped with a Phenomenex Kinetex C18 column (Table 4).
  a. Acquire data using a high throughput 5 min gradient in negative and positive ion modes – 2 runs per sample (Table 5).
    ***Note:*** We recommend running the full sample-randomized sequence in one polarity mode then again in the second polarity mode with several blank and QC injections between. The Vanquish UHPLC is coupled to a Thermo Q Exactive mass spectrometer via a heated electrospray ionization (HESI) source (Table 6). Users of this protocol are encouraged to optimize analytical and startup parameters given their instruments and research questions to generate the best possible data.

### Proteomics data acquisition parameters

**Timing: 5 h for the first 5 samples plus accompanying QC runs; add 48 min increments as needed per additional sample which includes time for accompanying QC runs**

45. Use liquid chromatography to separate the isolated peptides prior to MS acquisition.
  ***Note:*** We utilize an Evosep One system equipped with a Pepsep column, (150 mm inter diameter, 15 cm) packed with ReproSil C18 1.9 μm, 120 A resin (Table 7). The Evosep One system is coupled online to a Bruker timsTOF Pro mass spectrometer via a nano-electrospray ionization source.
  a. Analyse eluted peptides by high-resolution mass spectrometry in data-dependent acquisition mode (DDA) (Table 8).
    ***Note:*** We set the mass spectrometer to operate in Parallel Accumulation–Serial Fragmentation (PASEF) mode. We include below the settings utilized in this study as an example framework; users of this protocol are encouraged to optimize parameters given their instruments and equipment to generate the best possible data.
    ***Note:*** Other nanoLC and high-resolution mass spectrometer systems with similar capabilities may be used, including the Orbitrap and timsTOF series. For other instruments, parameters can be adjusted to suit the specific system in use.

### Metabolomics and stable isotope tracing data analysis

**Timing: 3+ h dependent on experiment complexity**

46. Process metabolomics mass spectra according to standard approaches.
  ***Note:*** With high throughput methods and carefully maintained UHPLCs, retention time alignment is not necessary. Our pipelines utilize El-Maven (Elucidata) for metabolite assignment against an in-house standard library of 1100 compounds (IROA Technologies).
  a. Normalize data for cell supernatants to the counts of the corresponding cell samples if such cell counts are not identical across the sample set.
47. Alternatively, perform stable isotope tracing analysis with El-Maven using automatic isotope detection options for the isotope(s) of interest (e.g., ^13^C and/or ^15^N).
  ***Note:*** The combination of high-resolution MS with narrow (5–10 ppm) peak windows in the chosen data analysis software are both necessary to distinguish isotopologues with very similar m/z, e.g. ^13^C_3_, ^15^N glutamate versus ^13^C_4_ glutamate.
  ***Note:*** Data analysis requires significant curation including an assessment of spectral properties for each isotopologue, knowledge of metabolic pathways, and assessment of QC runs to reduce the chance of a false positive identification.
  a. Normalize data for cell supernatants to the counts of the corresponding cell samples if such cell counts are not identical across the sample set.
    ***Note:*** Natural isotope correction should be applied to peak areas for ^13^C_1_, ^13^C_2_, and ^15^N_1_ peak areas to account for contributions made by the parent (i.e. unlabeled) metabolite. Such calculations can be performed in Microsoft Excel or similar software and consider the number of labeled atoms, the total number of the atom (e.g. N) in the molecule, and the natural abundance of ^13^C (1.1%) and/or ^15^N (0.4%).

### Lipidomics database searching and identification

**Timing: 2+ h dependent on experiment complexity and batch size**

48. Search lipidomics files against databases to identify and quantify lipids.
  a. Utilize LipidSearch (Thermo) which accepts.raw files generated from Thermo mass spectrometers.
  b. Search.raw files, one polarity per search, using mass tolerances appropriate for your instrumentation and application.
    ***Note:*** We recommend utilizing a mass tolerances of 5 ppm for precursor ions and 25 ppm for product ions.
    ***Note:*** Data files for QC runs and blank runs are included.
  c. When searches have completed, align search results by classifying files into their biological groups.
  d. Normalize data for cell supernatants to the counts of the corresponding cell samples if such cell counts are not identical across the sample set.
49. Alternatively, process lipidomics files using steps 46–47 to assess enrichment of the lipidome from stable isotope labeled substrates.

### Proteomics database searching and identification

**Timing: 2+ h dependent on experiment complexity and batch size**

50. Search proteomics spectra against database to quantify peptides and map to proteins.
  a. Extract MS/MS spectra from raw data files and convert to.mgf files using MS Convert (ProteoWizard, Ver. 3.0).
  b. Search resulting.mgf files against the Uniprot human database using a search engine such as Mascot.
    ***Note:*** We recommend utilizing Mascot v. 2.5 with the following parameters: mass tolerances ±15 ppm for parent ions, and ±0.04 Da for fragment ions. Trypsin specificity with 1 missed cleavage. Variable modifications: Met oxidation, protein N-terminal acetylation, Lys ^13^C acetylation, and peptide N-terminal pyroglutamic acid. Fixed modifications: Cys carbamidomethylation.
    ***Note:*** The latest version of the human proteome can be downloaded as a FASTA file from uniport.org
  c. View and validate search results using software such as Scaffold (v 5.0, Proteome Software, Portland, OR, USA) with FDR cutoff of 5%.
    ***Note:*** Peptide identifications are accepted if established at >95.0% probability as specified by the Peptide Prophet algorithm. Protein identifications are accepted if established at >99.0% probability and containing at least two identified unique peptides.

## Expected outcomes

This protocol enables consistent and reproducible preparation of iNSC monocultures for downstream isotopic tracing and multi-omics profiling. Upon successful completion of the culturing and tracing phases, users should expect robust and healthy iNSC monocultures with greater than 90% viability, approximately 70–80% confluency at the time of tracing, and uniform cellular morphology. The use of labeled substrates such as ^13^C_6_-Glucose or ^13^C_5_,^15^N_2_-glutamine should result in efficient isotopic enrichment of downstream metabolites and lipids in both intracellular and extracellular compartments, as later confirmed by mass spectrometry. Provided that recommended seeding densities and medium volumes are followed, each sample will yield sufficient biomass for multiomic extraction, along with consistent recovery of both conditioned medium and cells suitable for metabolomics and lipidomics workflows, with minimal contamination from serum-derived or exogenous metabolites. High-quality peptide samples are also expected for LC-MS/MS analysis, typically yielding 1–2 μg/μL after digestion from both cell lysates and conditioned media. Protein digestion efficiency should remain consistent, showing minimal missed cleavages when optimal trypsin conditions are applied (e.g., 1:50 enzyme-to-protein ratio); overnight digestion at 37°C is recommended for FASP, whereas S-Trap digestion generally requires only 2–6 h. Peptide recovery typically ranges from 70–90%, depending on protein input and cleanup strategy. Overall, this workflow supports high-confidence molecular characterization of iNSC monocultures across conditions through metabolomics, lipidomics, and proteomics, with reproducible identification and quantification of approximately 1,000–5,000 proteins in iNSC cell lysates using standard data-dependent acquisition (DDA) methods, and proportionally fewer proteins detected in conditioned media depending on sample complexity and input volume. Finally, the protocol is fully compatible with multi-omic integration, as protein samples are processed in parallel with metabolomic and lipidomic fractions derived from the same biological replicate, enabling comprehensive profiling of cellular responses.

## Limitations

The protocol was optimized for specific iNSC lines. Other iNSC lines or induced pluripotent stem cell-derived NSCs may require adjustment in seeding density, medium composition, or adaptation time to achieve comparable confluency and metabolic activity. Batch-to-batch variability in Matrigel coating or media components (e.g., B-27, GlutaMAX) can impact iNSC adhesion, growth kinetics, and metabolic responses. Timepoints for stable isotope tracing are affected by reaction kinetics and particular pathways of interest. We recommend including cell and cell supernatant samples that have not been exposed to stable isotope substrates for comparison and quality control of MS data. Selection of tracing timepoints should be performed based on pilot experiments and/or consultation with literature precedents for similar systems. Batch effects in mass spectrometry are significant, thus all samples to be compared should be analyzed on the same day within the same sequence using aliquoted high quality reagents from the same lot when possible and including process controls. Mass spectrometry has inherent limitations in dynamic range, which may underrepresent low-abundance proteins, especially in complex samples such as whole-cell lysates. Optimization of digestion time, enzyme concentration, and peptide desalting conditions is important for reproducible results.

## Troubleshooting

### Problem 1

Cells detach during medium change (step: iNSC preparation for isotopic tracing).

### Potential solution

Cell detachment may be caused by incomplete cell adhesion or overly harsh aspiration. To keep these risks at a minimum, use wide-bore pipette tips and gently aspirate medium from the edge. Additionally, ensure cells are plated at least 24 h before tracing.

### Problem 2

Insufficient sample yield for MS (step: medium and cell collection following isotopic tracing).

### Potential solution

This may be a result of low cell density or inefficient harvesting. Potential solutions include scaling up the well/flask size used per sample or pooling replicates if needed. Ensure complete removal of cells by checking the well/flask under a microscope following collection. Complete removal of the PBS supernatant following cell pelleting and the prompt snap-freezing of cell pellets are also crucial.

### Problem 3

Contamination of cell pellets with medium (step: medium and cell collection following isotopic tracing).

### Potential solution

After removing tracing medium, wash cells 2–3× with ice-cold PBS and carefully aspirate to minimize carry-over before collection. All wash buffer must be removed before pellets are snap frozen.

### Problem 4

Minimal isotopic enrichment observed (step: iNSC preparation for isotopic tracing).

### Potential solution

This often results from the cells not taking up adequate amounts of tracer or from attempting stable isotope tracing in the presence of unlabeled substrate (i.e., adding ^13^C_6_ glucose to media containing ^12^C_6_ glucose). Pilot experiments can often fine tune the amounts and timing of stable isotope labelled substrates in cell culture. Concretely, time-course pilot experiments help track labeling dynamics and identify the optimal time point for maximal enrichment.

### Problem 5

Insufficient peptide yield for mass spectrometry (step: sample processing for proteomics—cells).

### Potential solution

• Ensure the samples contain approximately 5% SDS before proceeding with acidification and loading onto the S-Trap. SDS is essential for forming colloidal protein particulates that enable efficient protein trapping on the S-Trap matrix.
• Acidification to a final concentration of ∼1% phosphoric acid is necessary to induce protein precipitation in the presence of SDS. Confirm that the SDS-containing lysate is thoroughly acidified and that the final solution is highly acidic prior to mixing with the S-Trap binding buffer.
• If the acidified lysate is centrifuged before being added to the S-Trap unit, protein particulates may be pelleted and not enter the column. Ensure complete and immediate transfer of the acidified lysate/S-Trap buffer mixture into the S-Trap unit without prior centrifugation.
• Low digestion efficiency can result from insufficient enzyme levels, suboptimal temperature, or short digestion times. Optimize trypsin or protease concentration (e.g., 1:50 enzyme-to-protein ratio), ensure proper digestion temperature (typically 37°C), and allow sufficient time (e.g., 6 h for S-Trap digestion, overnight for FASP). If digestion is incomplete, consider multi-enzyme strategies (e.g., trypsin combined with LysC and collagenase) which may improve efficiency. However, keep in mind that multi-enzyme digestion can complicate downstream bioinformatics analysis and should be planned accordingly.
• If the S-Trap column is not sealed properly, the digestion buffer may evaporate, reducing enzyme activity. Securely seal the top cap of the S-Trap column during incubation to prevent evaporation.

### Problem 6

Carryover between MS runs (step: sample processing for proteomics—cells).

### Potential solution

Implement thorough wash protocols using strong solvents such as 80% acetonitrile with 0.1% formic acid. Extend the needle and loop wash cycles in the autosampler method and include blank injections between samples to detect and monitor residual signal. Regularly inspect and clean critical LC components, and replace consumables (e.g., wash lines, needle seats) as needed to maintain system hygiene.

### Problem 7

Variability within biological groups in omics data sets (step: medium and cell collection following isotopic tracing).

### Potential solution

Often this results from inaccurate cell counts, treatments which impact cell size, or, in the case of metabolomics or lipidomics, differential amounts of wash buffer remaining on top of frozen cell pellets leading to variable and impactful ion suppression. To address issues with cell counts or cell size, we recommend normalization to protein or nucleic acid concentration. Disentangling varied amounts of wash buffer from biological changes in omics data is not straightforward and generally requires repreparing cell pellets for analysis.

## Resource availability

### Lead contact

Further information and requests for resources and reagents should be directed to and will be fulfilled by the lead contact, Prof. Angelo D’Alessandro (angelo.dalessandro@cuanschutz.edu).

### Technical contact

Technical questions on executing this protocol should be directed to and will be answered by the technical contact, Rosana-Bristena Ionescu (rbi24@cam.ac.uk).

### Materials availability

This protocol did not generate new materials.

### Data and code availability

• Proteomics data have been deposited onto MassIVE:MSV000095283.
• The metabolomics (steady state and stable isotope tracing) and lipidomics data described in this study are available at the NIH Common Fund’s National Metabolomics Data Repository (NMDR) website, the Metabolomics Workbench, https://www.metabolomicsworkbench.org with the following study IDs: ST003331, ST003332, ST003328, and ST003330.

## Acknowledgments

The authors wish to acknowledge L. Ionescu, V. Pappa, G. Pluchino, O. Hruba, M. Sciacovelli, A. Speed, and A. Tolkovsky for technical and intellectual inputs throughout this study.

This research was supported by the Ferblanc Foundation G112716 (to S.P. and A.M.N.), Catalyst Award from the UK Multiple Sclerosis Society H160 (to S.P. and C.M.W.), 10.13039/100000890National Multiple Sclerosis Society Research Grant RG 1802-30200 (to S.P. and L.P.-J.), Bascule Charitable Trust RG98181 (to S.P.), 10.13039/100008191Wings for Life RG 82921 (to S.P. and L.P.-J.), and the 10.13039/100007366Fondazione Italiana Sclerosi Multipla FIMS 2018/R/14 (to S.P. and L.P.-J.) and 2022/R-Single/011 (to S.P.). R.-B.I. is supported through an MRC-DTP and Cambridge Trust studentship and consumable award (RG86932) and St. Edmund’s College Tutorial Award. A.M.N. is the recipient of a European Committee for Treatment and Research in Multiple Sclerosis (ECTRIMS) Postdoctoral Research Fellowship Exchange Program fellowship (G104956) and is supported through a UK Multiple Sclerosis Society Centre Excellence grant (G118541). P.P. is supported through an MRC-DTP and Cambridge Trust PhD studentship and consumable award (RG86932) and Queen’s College Tutorial Award. L.P.-J. was supported by a 10.13039/100007366Fondazione Italiana Sclerosi Multipla FIMS and Italian Multiple Sclerosis Association AISM Senior research fellowship financed or co-financed with the “5 per mille” public funding cod. 2017/B/5, a 10.13039/100010269Wellcome Trust Clinical Research Career Development Fellowship (G105713), and National Multiple Sclerosis Society Research Grant RFA-2203-39318. C.M.W. is the recipient of a National Multiple Sclerosis Society (USA) Post-doctoral fellowship (FG-2008-36954). F.E. is supported through the 10.13039/501100002428Austrian Science Fund (FWF) (Special Research Program F7804-B; I 4791; TAI 801) and EJP European Joint Programme on Rare Diseases (I 5184).

## Author contributions

Conceptualization, R.-B.I., J.A.R., A.D., and S.P.; methodology, R.-B.I., J.A.R., A.M.N., M.D., D.S., P.P., C.M.W., M.S.C., L.P.-J., C.F., A.D., and S.P.; investigation, R.-B.I., J.A.R., A.M.N., M.D., D.S., P.P., C.M.W., and L.P.-J.; writing – original draft, R.-B.I., J.A.R., and M.D.; writing – review and editing, R.-B.I., J.A.R., M.D., A.M.N., C.M.W., L.P.-J., A.D., and S.P.; funding acquisition, R.-B.I., A.M.N., P.P., C.M.W., L.P.-J., A.D., and S.P.; resources, M.S.C., and F.E.; supervision, C.F., A.D., and S.P.

## Declaration of interests

S.P. is the founder, CSO, and shareholder (>5%) of CITC Ltd. and Chair of the Scientific Advisory Board at ReNeuron plc. A.D. is a founder of Omix Technologies Inc. and Altis Biosciences LLC and an advisory board member for Hemanext Inc. and Macopharma Inc.

## References

[1] Ionescu R.B., Nicaise A.M., Reisz J.A., Williams E.C., Prasad P., Willis C.M., Simões-Abade M.B.C., Sbarro L., Dzieciatkowska M., Stephenson D. (2024). Increased cholesterol synthesis drives neurotoxicity in patient stem cell-derived model of multiple sclerosis. Cell Stem Cell.

